# Data on electrical properties of nickel modified potassium polytitanates compacted powders

**DOI:** 10.1016/j.dib.2015.05.010

**Published:** 2015-05-28

**Authors:** V.G. Goffman, A.V. Gorokhovsky, N.V. Gorshkov, F.S. Fedorov, E.V. Tretychenko, A.V. Sevrugin

**Affiliations:** Yuri Gagarin State Technical University of Saratov, 77 Politechnicheskaya Street, 410054 Saratov, Russia

**Keywords:** Modified potassium titanates, Permittivity, Conductivity, Dielectric losses

## Abstract

Potassium polytitanates are new promising type of ferroelectric ceramic materials with high ionic conductivity, highly polarizable structure and extremely high permittivity. Its structure is formed by [TiO_6_] octahedral units to layers with mobile potassium and hydroxonium ions in-between. The treatment in solutions containing nickel ions allows forming heterostructured materials which consist of potassium polytitanate particles intercalated by Ni^2+^ ions and/or decorated by nickel oxides NiO_*x*_. This modification route is fully dependant on solution pH, i.e. in acidic solutions the intercalation process prevails, in alkaline solutions potassium polytitanate is mostly decorated by the oxides. Therefore, electronic structure and electrical properties can be regulated depending on modification conditions, pH and ions concentration. Here we report the data on electric properties of potassium titanate modified in nickel sulfate solutions at different pH.

**Table:** 

Subject area	Materials science
More specific subject area	Solid state electrolytes. Ceramics
Type of data	Table, figures
How data was acquired	Energy-Dispersive X-ray Fluorescence Spectrometer BRA-135F
	STD Q600 TA Instruments (Thermogravimetric Analysis)
	Impedance-meter Novocontrol Alpha-AN
Data format	Analyzed
Experimental factors	Electrical properties were investigated by impedance spectroscopy in the frequency range 10^-2^ Hz–10^6^ Hz applying periodic signal 50 mV
Experimental features	Potassium titanates modification in aqueous solution of Ni salts at different pH; compacting; thermal treatment
Data source location	Department of Chemistry, Yuri Gagarin State Technical University of Saratov, Russia
Data accessibility	Data are available with this paper

**Value of the data**

• The data highlights a soft chemistry method to regulate structure and electric properties of layered ceramic materials.
• These data show that modification of potassium polytitanate powders in aqueous solutions of nickel sulfate allows regulating the materials electric properties (permittivity, ionic and electronic conductivity, dielectric losses).
• The data points on importance of adsorbed water to electrical properties of potassium titanates and its involvement in charge transfer processes, i.e. conductivity of the titanates.

## 1 Experimental design, materials and methods

The obtained parent and modified potassium polytitanate (PPT) powders were studied by XRD using ARL X׳TRA Thermo Scientific what confirmed its (X-ray) amorphous structure. The amorphous structure of the titanates remains unchanged up to 500–600 °С [1]. Chemical composition was confirmed by Energy-Dispersive X-ray Fluorescence Spectrometer BRA-135F (Table 1).

Water contents in the investigated powders were measured using thermo-gravimetric method (STD Q600 TA Instruments). According to the obtained data and our previous results physically adsorbed water is desorbed from PPT powders at 100–220 °C, whereas structural water (located in the interlaminar space of PPT particles) is being removed at 350–500 °C [1].

Impedance spectroscopy was conducted in the frequency range 10^−2^–10^6^ Hz applying periodic signal with amplitude 50 mV (impedance-meter Novocontrol Alpha-AN). Electrical contacts were realized by claying metal wires with conductive paste Contactol-K13 to the round sides of the compacts. The rest surface was isolated by epoxy compound Pentelepst 1143-A. Obtained data was used to calculate conductivity *σ,* dielectric dissipation factor, *tan*(*δ*), and complex permittivity *ε*^⁎^ [2].

## 2 Material preparation

The PPT powder was produced by the treatment of powdered TiO_2_ (anatase, Aldrich 99%+, average size of particles ca. 7 μm) in the molten mixture of KNO_3_ and KOH at 500 °C for 2 h according to [1]. The TiO_2_:KOH:KNO_3_ weight ratio was 1:1:8. The synthesized potassium polytitanate was carefully washed with distilled water three times (1 dm^3^ of H_2_O per 100 g of the product for each washing), filtrated (Whatman paper No 40) and dried at 60 °C in an oven to obtain the parent (X-ray) amorphous PPT powder characterized with the TiO_2_:K_2_O molar ratio 4:1.

The parent PPT powder was modified according to the following route [3,4]: 30 g of powder was put in a glass which then was filled up with 200 ml of 0.001 M nickel sulfate solution. pH of the obtained dispersion was adjusted by additions of 10% H_2_SO_4_ or 10% KOH to acidic (pH=3.0), neutral (6.5) or alkaline (9.0). The obtained suspension was stirred 4 h and then the powder was separated and dried at 60 °C.

Modified PPT powders were compacted to density 2.2 g/cm^3^ in disks of 8 mm of diameter and 2.0±0.1 mm of thickness. Electric properties were studied with the parent compacts and with similar specimens obtained after the thermal treatment of parent compacts in the oven at 160 °С for 6 h for total desorption of the physically adsorbed water. All the measurements were conducted at room temperature.

## 3 Chemical composition data

The powders modified in acidic solution show a relatively low content of NiO. Modification in alkaline solution results in high amount of both NiO and K_2_O in the PPT powder. Particularly, K_2_O content is high, as a consequence of a low ion-exchange rate. The powders obtained in neutral solution (рН=6.5, close to isoelectrical point for nickel sulfate aqueous solution, 0.001 M) show moderate values for NiO and K_2_O which are higher than in acidic solution but lower than in alkaline media [3].

## 4 Data on electrical properties of the PPT compacts modified in nickel sulfate solution

**Fig. 1:**
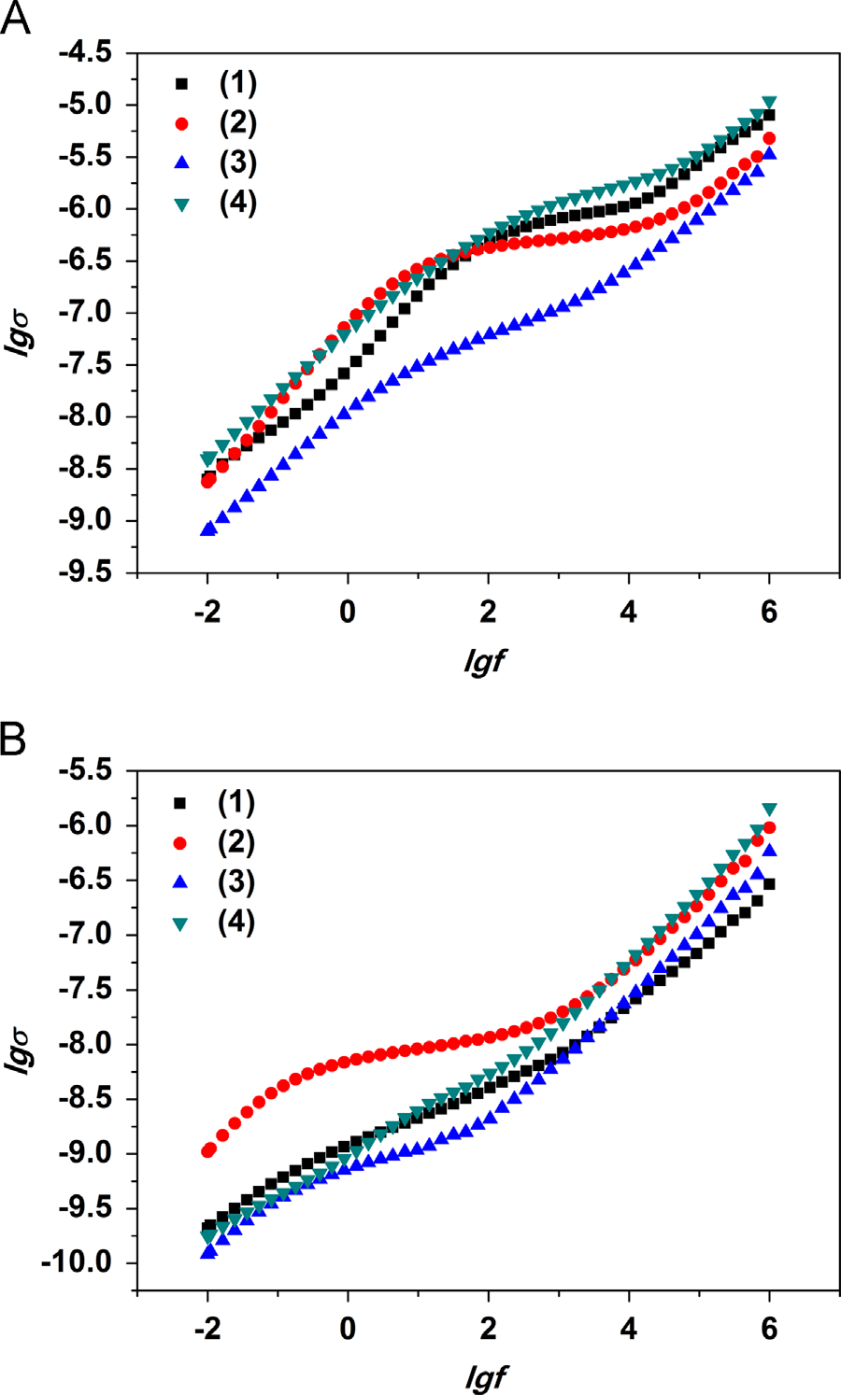
Bode conductivity plots for compacted parent PPT (1) and PPT modified in solution containing nickel ions at рН=3.0 (2), 6.5 (3) и 9.0 (4) before (A) and after the thermal treatment (B).

**Fig. 2:**
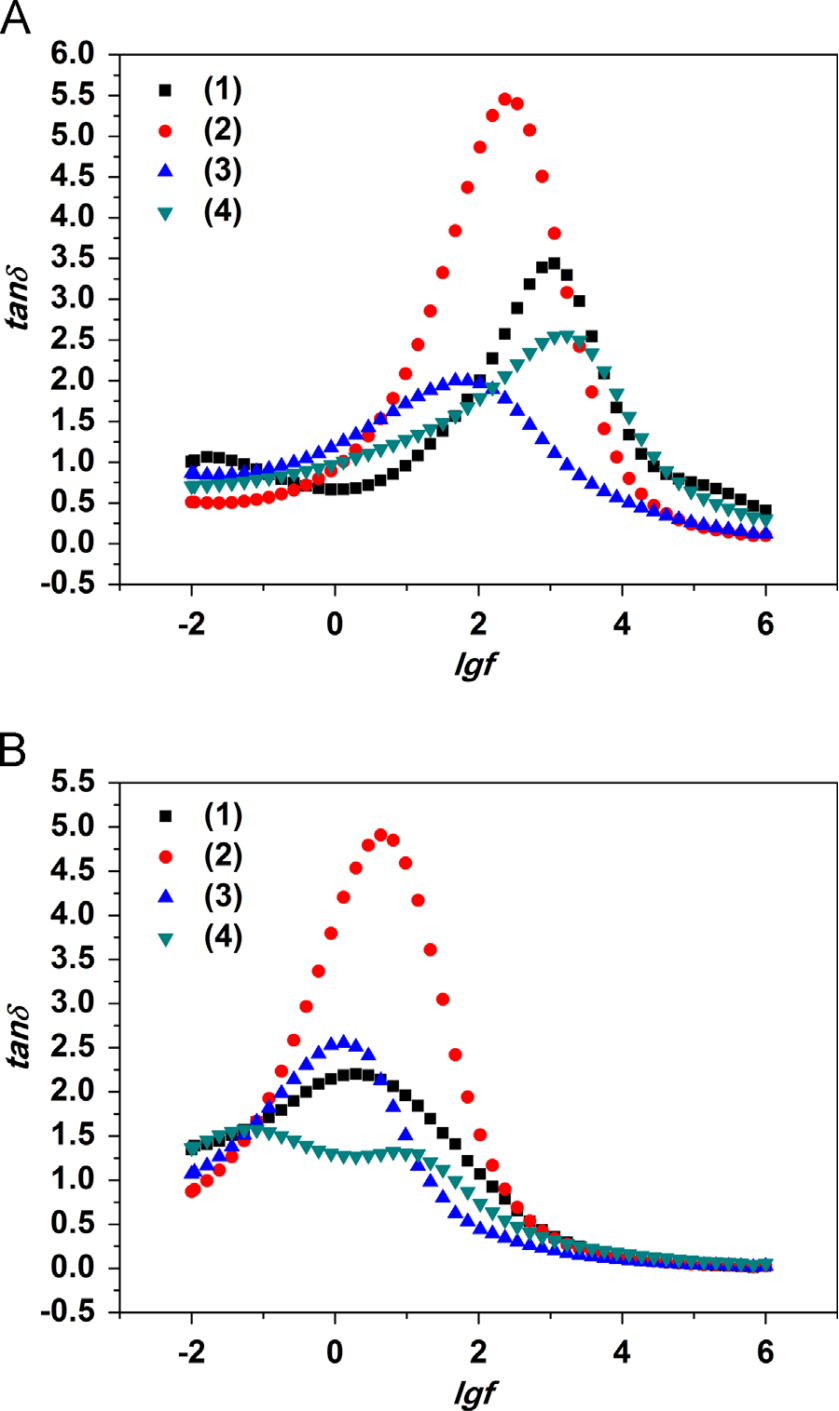
Bode *tan*(*δ*) plots for compacted parent PPT (1) and PPT modified in solution containing nickel ions at рН=3.0 (2), 6.5 (3) и 9.0 (4) before (A) and after the thermal treatment (B).

**Fig. 3:**
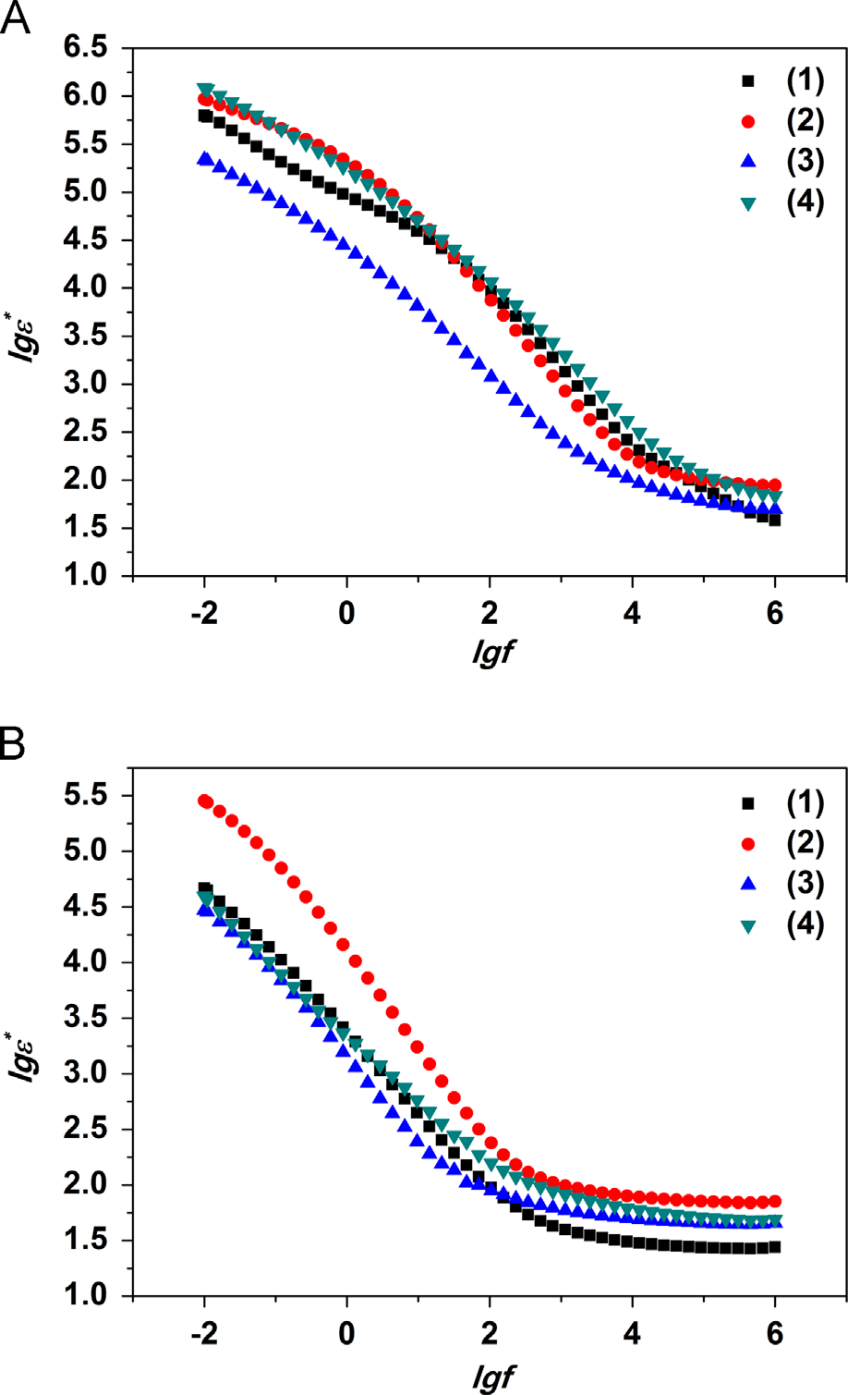
Bode complex permittivity plots for compacted parent PPT (1) and PPT modified in solution containing nickel ions at рН=3.0 (2), 6.5 (3) и 9.0 (4) before (A) and after the thermal treatment (B).

Bode plots for modified PPT compacts before and after the thermal treatment are presented in Figs. 1–3.

The conductivity data show a linear change at low frequencies (Fig. 1). By extrapolating it to the frequency 10^−2^ Hz one might assess dc conductivity, *σ*_*dc*_, in dependence on composition of particular compact before or after the thermal treatment. Compacts before the thermal treatment (containing physically adsorbed water) show dc-conductivity similar to the one for the parent PPT (~5·10^–9^ Sm/cm) [5]. However, compacts modified at pH=6.5 show lower conductivity *σ*_*dc*_ ~10^–9^ Sm/cm. After removal of adsorbed water from the PPT/Ni modified at pH 6.5 its conductivity *σ*_*dc*_ remains the same 10^–9^ Sm/cm. Though, for PPT/Ni (pH=3.0) the conductivity *σ*_*dc*_ drops to ~10^–10^ Sm/cm.

The highest ac conductivity, *σ*_*ac*_, at ~10^6^ Hz is found for the parent PPT powder compacts and the compacts of PPT modified in alkaline solution (*σ*_*ac*_ ~10^–5^ Sm/cm), i.e. with relatively high К_2_О content. After the thermal treatment and consequent removal of the adsorbed water conductivity shows a change of about an order (~10^–6^ Sm/cm) for the modified PPT compacts and the drop is 1.5 orders (~5·10^–7^ Sm/cm) for the parent PPT compacts.

Data on *tan*(*δ*) shows a presence of several relaxation peaks. The peaks might be attributed to two relaxation processes with relaxation times of 6–60 s and 0.1–1 s respectively. The first peak is presumably due to proton transfer along grain boundaries of the compacted particles either for pure PPT or for their modified specimens, which are covered with physically absorbed water. This correlates with the results for *tan*(*δ*) obtained for the samples before the thermal treatment. The second peak, appeared at lower frequencies, should be due to proton transfer via structural water localized in the interlayer space of PPT particles. The potassium polytitanate compacts modified at low pH (рН=3.0) possess the highest values of *tan*(*δ*) in the region 1–10^2^ Hz while a product obtained by modification in neutral and alkaline solutions and, therefore, less hydrated, shows the lowest *tan*(*δ*) values. Correspondingly, conductivity of the compacts PPT/Ni (pH=3.0) is maximal at lower frequencies. Bode plots of dielectric dissipation factor for compacted PPT powders with high K_2_O content (parent PPT and PPT/Ni (pH=9.0)) (Fig. 2A) shows characteristic peaks at ~10^3^ Hz. At the same time, for the compacts with low K_2_O content PPT/Ni (рН=6.5 and рН=3.0) the peak is at ~10^2^ Hz. A small maximum is also observed at 0.01 Hz as well for PPT compact prepared from parent powder.

After the thermal treatment, i.e. removal of adsorbed water (Fig. 2B) maxima of the peaks of *tan*(*δ*) for all the compacts are shifted to low frequency region (~0.5 Hz), with maximum intensity refferred to a peak for PPT modified in acidic solution. Two peaks are observed for PPT/Ni (pH=9.0) at 10^−1^ Hz and 10 Hz.

All the investigated compacts can be characterized by a high permitivity (~10^6^) at low frequencies and permitivity ~10^2^ in high frequencies region. It is worth highlighting that the permitivity of PPT compacts modified in the neutral solution is lower comparing to the other compacts and equals 4·10^5^ at low frequencies. After removal of physically adsorbed water it decreases to (5–7)·10^4^ following the same trend for the studied compacts. At the same time for PPT/Ni obtained in acidic solution, permitivity remains at value of 5·10^5^. At high frequencies all modified PPT compacts show higher permivity values comparing to parent PPT; the highest permitivity (95) is ascribed for PPT/Ni compact modified in acidic solution.

The best polarization properties (the highest permittivity) are confirmed for the compacts obtained from PPT powders by their treatment in aqueous solutions of nickel sulfate at pH=6.5. This product is characterized with complicated structure consisting of PPT particles partially intercalated with Ni^2+^ ions and decorated by NiO_*x*_ nanoparticles.

This data shows that modification of PPT powders in aqueous solutions of nickel sulfate allows obtaining the materials with high permittivity in low-frequency region as well as with low ionic and electronic conductivity. The compacts produced with the PPT/Ni (pH=3.0) powder thermally treated at 160 °C are characterized with high value of permittivity which remains up to the temperature of 350–400 °C while content of structural water in the composition of this powder does not change significantly.

## Figures and Tables

**Table 1:** Chemical composition (in mass%) of the PPT powders modified in different conditions.

**Conditions**	**X-ray fluorescence analysis data**					**TGA data**	
	**K**_**2**_**O**	**TiO**_**2**_	**NiO**	**Al**_**2**_**O**_**3**_	**SiO**_**2**_	**Physically adsorbed H**_**2**_**O (25** °**C<*****Т*****<220** °**C)**	**Structural H**_**2**_**O (350** °**C<*****Т*****<500** °**C)**
(рН=3.0)	0.7	95.9	1.2	1.2	1.0	8.3	4.2
(рН=6.5)	1.8	82.2	15.2	0.2	0.6	7.6	2.5
(рН=9.0)	4.7	73.4	20.5	0.8	0.6	4.8	1.8
Parent PPT powder (рН=10.1)	19.5	79.8	–	–	0.7	7.8	3.1
